# Mammary Fat Can Adjust Prolactin Effect on Mammary Epithelial Cells via Leptin and Estrogen

**DOI:** 10.1155/2009/427260

**Published:** 2009-11-12

**Authors:** Yonatan Feuermann, Sameer J. Mabjeesh, Avi Shamay

**Affiliations:** ^1^Institute of Animal Science, Agricultural Research Organization, The Volcani Center, P.O. Box 6, Bet Dagan 50250, Israel; ^2^Department of Animal Science, Faculty of Agriculture, Food and Environmental Quality Sciences, Hebrew University of Jerusalem, P.O. Box 12, Rehovot 76100, Israel

## Abstract

Leptin, like estrogen, is one of the endo/paracrine factors, which are synthesized in and secreted from mature adipocytes. The roles of the mammary fat pad and mammary adipocytes in the initiation of lactation are not clear. In this study, we showed that combination of prolactin, leptin and estrogen elevated the expression of the milk protein beta-lactoglobulin. We also showed that after prolactin stimulate the secretion of leptin from the mammary fat, leptin upregulated the expression of estrogen receptor alpha in the mammary epithelial cells. Also, prolactin affected aromatase mRNA expression in the bovine mammary fat and we demonstrated that leptin and prolactin can affect cholesterol secretion from explants in culture to the medium. Therefore, we suggest that prolactin initiates estrogen expression (as represented by aromatase mRNA) in the mammary fat pad, whereas leptin stimulates estrogen receptor alpha expression in the mammary epithelial cells. We hypothesize that leptin and estrogen, secreted from the mammary fat regulate lactation after stimulation of prolactin.

## 1. Introduction

Traditionally, the function of the adipose tissue is regarded as energy storage. However, recent studies showed that the adipose tissue is a highly active endocrine organ, which synthesizes and secretes a large number of proteins, such as leptin, resistin, adiponectin, angiotensinogen, and many others. Some of these proteins induce insulin resistance, others play a role in glucose and lipid metabolism or even as a mediators of the immune system [1]. The mammary fat pad, was considered as an inert matrix, however it is believed to be a hormone-producing tissue [2–4]. Recent evidence suggests that the adipocytes-epithelial interaction is critical for mammary duct growth and morphogenesis [5, 6]. Generally, estrogen is required for growth and morphogenesis of the mammary gland in rodents [7] and bovine [8], and it is also essential for mammary epithelial proliferation and differentiation [9]. Estrogen receptor alpha mRNA expression is abundant in the mammary gland of non-pregnant heifers [10, 11], lactating cows [11] and human [12]. It was shown that the bovine mammary gland secretes estrogen during the last week of pregnancy [13], and that estrogen effects are mediated mainly by estrogen receptor alpha in the mammary gland. Estrogen receptor alpha expression is limited to the bovine mammary epithelial cells during pregnancy, lactation and regenerative involution [11]. Transplantation of epithelial and stroma cells from estrogen receptor alpha-null mice and wild-type mice demonstrated that estrogen responsiveness was mediated by paracrine factors, and that estrogen receptor alpha responses varied with the developmental stage of the mammary gland [14]. 

The role of leptin in the regulation of bovine lactation is not clear. Leptin has been found in mammary adipocytes of sheep during the early phase of pregnancy, and in mammary epithelial cells during lactation [15]. The leptin receptor is a member of the class I cytokine receptor family, and is present in five different spliced forms: Ob-Ra, Ob-Rb, Ob-Rc, Ob-Rd, and Ob-Re [16]. The tissue distribution of leptin receptor isoforms in bovine is not well established. The expression of Ob-Ra has been reported previously only in the adrenal gland [17], pituitary gland, liver and spleen in cattle [18], and in the lactating ovine mammary gland [19]. Previously we demonstrated that leptin alone had no effect on alpha casein gene expression and fatty acid synthesis in bovine mammary explants, nonetheless in the presence of prolactin, leptin up-regulated alpha casein and beta lactoglobulin expression, and fatty acid synthesis [20]. Leptin is believed to be one of the markers of matured adipocytes [1]. During the terminal phase of differentiation to mature adipocytes, activation of the transcriptional cascade leads to increased metabolic activity that converts the adipocyte to a highly specialized endocrine cell, in which one of the factors is leptin synthesis and secretion [21]. The lactogenic hormone prolactin is one of the factors that stimulate adipogenesis in preadipocyte cell lines and has variable lipolytic and lipogenic effects in mature adipose tissue [22]. Prolactin had been assumed to be involved in mammogenesis [6]. Prolactin receptor has been expressed in the mammary gland fat pad in humans [23], and ovine mammary parenchyme [24]. Recent studies showed that after 5-6 weeks of lactation, the plasma levels of prolactin begins to rise and short time after, leptin concentration increased as well [25]. In the current study we used three different culture systems: (a) primary cell culture that contains mostly mammary epithelial cells, (b) hind udder fat explants that contain mostly fat cells, and (c) mammary explants that are the closest in vitro system to the udder. These in vitro systems allowed us to investigate whether prolactin interacts with the bovine mammary fat-driven estrogen and leptin, and to look for the effects of leptin and estrogen on lactogenic parameters in mammary gland cells and explants, after stimulation with prolactin.

## 2. Materials and Methods

### 2.1. Mammary Tissue Collection

Mammary tissue was obtained from Holstein cows at the slaughterhouse, 2 to 5 months after the onset of lactation. The Israeli Ministry of Agriculture approved all procedures in accordance with Israeli regulations for animal experimentation.

### 2.2. Materials

M-199, penicillin, streptomycin, and Fungi zone (amphotericin B) were obtained from Bet Haemek, Israel. Bovine insulin, cortisol, estrogen and ovine prolactin were purchased from Sigma (St. Louis, MO). Bovine recombinant leptin and leptin antagonist were generously given as a gift from Prof A. Gertler [26].

### 2.3. Mammary Explants Culture

Mammary tissue were obtained from 3–5 cows at the slaughterhouse, and were transferred into medium M-199, containing 100 U penicillin, 100 *μ*g streptomycin, 0.25 *μ*g Fungi zone, and 1 *μ*g insulin (per millliliter). Explants were prepared as previously described [27]. Twenty explants, 50–80 mg total weight from a one udder quarter, were placed on an impregnated lens paper floating in 5 mL of M-199 medium, and cultured at 37°C in a medium supplemented with insulin at 1 *μ*g mL^−1^, cortisol at 0.5 *μ*g mL^−1^ and prolactin at various concentrations (IFP). Or in medium supplemented only with insulin at 1 *μ*g mL^−1^ and cortisol at 0.5 *μ*g mL^−1^ (IF). Leptin and leptin antagonist were added according to the experimental protocol. The medium was changed every 24 hours, for 5 days. 

### 2.4. Fat Explants Cultures

Mammary fat tissue from the hind udder portion was taken aseptically from cows at the slaughterhouse and transferred into medium M-199 containing 100 U penicillin, 100 *μ*g streptomycin, 0.25 *μ*g Fungi zone, and 1 *μ*g insulin (per milliliter), as previously described [28]. Fat tissue explants from a one udder quarter, were cultured, floating in 5 mL of M-199 medium, at 37°C in medium supplemented with insulin at 1 *μ*g mL^−1^, cortisol at 0.5 *μ*g mL^−1^ and prolactin at various concentrations, or in medium supplemented only with insulin at 1 *μ*g mL^−1^ and cortisol at 0.5 *μ*g mL^−1^ (basal medium). Leptin and leptin antagonist were added according to the experimental protocol. The medium was changed every 24 hours, for 5 days. 

### 2.5. Fat-Epithelial Co-Culture

Bovine mammary-gland tissue was transferred to the laboratory, cut into small pieces and placed in a 500-mL Erlenmeyer flask containing M-199 medium supplemented with collagenase at 1 mg mL^−1^, hyaluronidase at 1 mg mL^−1^ and bovine insulin at 1 *μ*g mL^−1^, at a ratio of 10 mL of supplemented medium to 1 g of tissue. The culture was swirled at 100 rpm in a gyrating water bath at 37°C for 3 to 4 hours. During this period, tissue fragments were further dissociated by occasional aspiration through a 10-mL pipette with a large orifice. One hour before termination of the enzymatic digestion, 0.04% DNase was added at 0.5 mL per 100 mL of digest. At the end of the incubation, the suspension was passed through a Nitex filter (200 *μ*m) and the cells were washed three to five times with M-199. The cells were grown in a 10-mm plastic dish in DMEM/F-12 (HAM) 1 : 1, supplemented with 10% fetal calf serum (FCS), and (per milliliter) 1 *μ*g insulin, 10,000 U penicillin, 10 mg streptomycin, 0.025 mg Fungi zone and 0.5 mg L-glutamine, in a humidified atmosphere of 5% CO_2_/95% air at 37°C. The medium was changed at 48-h intervals until the end of the experiment. The cells were grown to confluence, dispersed with 0.05% trypsin, transferred to new plates, and stored at −70°C in 10% DMSO and 20% FCS. The cells used in these experiments underwent no more than six transfers. Once the experiment began, fat explants were prepared as described in the Explants Culture section and were added to the primary mammary cell culture dish for 48 hours.

### 2.6. Western Blot Analysis

Western blotting was carried out according to Schuler et al. [29] with the following modifications. Samples (40 *μ*g protein in 20 *μ*l buffer) were boiled in 3x_loading buffer (10 mM Tris–HCl, pH 6.8, including 3% SDS, 5% mercaptoethanol, 20% glycerol and 0.6% bromophenol blue for 3 minutes, separated on 12% Bis-Tris gel under reducing conditions for 1 hour at 190 V, and transferred to nitrocellulose membranes (Millipore PVDF, 0.45 *μ*m; Millipore, Bedford, USA). For blocking, membranes were incubated overnight in PBS with 0.05% Tween-20 (PBS-T) with 1% non-fat dry milk. The membranes were then incubated in PBS-T and incubated for 75 minutes with the respective primary antibody, ER-alpha rabbit polyclonal IgG (dilution 1 : 200) against epitope corresponding to amino acids 2–185 mapping at the amino terminus of human ER alpha (no. sc 7207; Santa Cruz Biotech, Heidelberg, Germany). After washing in PBS-T, the membranes were incubated with the horseradish peroxidase-linked secondary goat anti-mouse or goat anti-rabbit immunoglobulin antibody (no. sc 7207; Santa Cruz Biotech) for 45 minutes. At the end, they were again washed in PBS-T, incubated in enhanced chemiluminescence reagents (ECL Western Blotting Analysis System; Amersham Pharmacia Biotech, Freiburg, Germany) for 30 s or 1 minute. Blue Plus 2 Pre-Stained Standard (Novex, San Diego, CA, USA) was used as the molecular size marker.

### 2.7. Total Cholesterol

In order to determine the total cholesterol secretion by the explants to the medium, eight replicates of each treatment were collected to form a total of 40 mL of medium. The medium was lyophilized and dissolved in a chloroform : methanol 2 : 1 (v/v) solution. Total cholesterol content was determined after saponification of 100 *μ*l of concentrated medium. After extraction in petroleum ether, colorimetric measurements were done with a modified Lieberman–Buchard reaction, as described previously [30]. 

### 2.8. Isolation of RNA

Total RNA was isolated from the mammary tissue by the acid guanidinium thiocyanate phenol-chloroform extraction method [31]. Following its isolation, the RNA was kept at −70°C pending analysis by RT-PCR or real-time PCR (rt-PCR).

### 2.9. Reverse Transcription (RT)

Total RNA (1 *μ*g) was reverse-transcribed in a final volume of 20 *μ*l containing: 50 mM Tris-HCl (pH 8.3), 75 mM KCl, 5 mM MgCl_2_, 10 mM dithiothreitol, 0.5 mM deoxy-NTP (dNTP), 0.5 *μ*g oligo-dT/Hexamer primers (Promega, Madison, WI), and 1 U avian myeloblastosis virus (AMV) reverse transcriptase (Promega). The reaction temperatures were 42°C for 1 hour and 95°C for 10 minutes.

### 2.10. Real-Time PCR (rt-PCR)

RNA samples were reverse-transcribed as described. Samples were analyzed by rt-PCR with the ABI Prism 7000 system (Applied Biosystems, Foster City, CA). A 5-*μ*l sample of cDNA was amplified for 40 cycles with 3 *μ*l of the following primers at a final concentration of 0.5 *μ*M: ER-alpha (sense 5′-AGG GGA GCT CCT ATT TGC TCC-3′, antisense 5′-CGG TGG ATG TGG TCC TTC TCT-3′); beta-lactoglobulin (sense 5′-ATC CCT GCG GTG TTC AAG AT-3′, antisense 5′-CCA TGC AGA CGA GCA GGT ACT-3′); aromatase primers (sense 5′-CCG GTC TCT GGT CTC GTC TG-3′, antisense 5′-TTC TGA GAC GCT TCC ACG TG-3′); and as a normalizing control, bovine ribosomal 18S (sense 5′-CGG CTA CCA CAT CCA AGG AA-3′, antisense 5′-GGG CCT CGA AAG AGT CCT GT-3′). The reaction was carried out in a final volume of 50 *μ*l, comprising 16 *μ*l of SYBR Green (Applied Biosystems), 3 *μ*l of 5 *μ*M primer mix, 3 *μ*l of 5 *μ*M reverse primer, 5 *μ*l of cDNA template and 23 *μ*l of deionized water, for 40 cycles of 1 minute of denaturation at 95°C, 2 minutes of annealing at 60°C, and 1 minute of extension at 72°C. The amplified PCR product was analyzed with ABI Prism 7000 software (Applied Biosystems). At the end of the rt-PCR run, a melting curve was determined to verify the presence of a single amplicon. The analysis of rt-PCR was preformed as previously described by feuermann et al. [28].

### 2.11. Statistical Analysis

All statistical analyses were carried out with the JMP 5.0.1 statistical package from SAS. The results of the explants experiments, leptin secretion and expression experiments were compared by one-way ANOVA. The results of the co-culture experiments were compared by means of Student's *t*-test [32] after one-way ANOVA. In all the experiments the control was considered as the basal treatment. (medium containing insulin at 1 *μ*g mL^−1^ and cortisol at 0.5 *μ*g mL^−1^).

## 3. Results

### 3.1. Effects of Leptin, Prolactin and Estrogen on Beta-Lactoglobulin Expression in Explants from Mammary Glands of Lactating Cows

**Figure 1:**
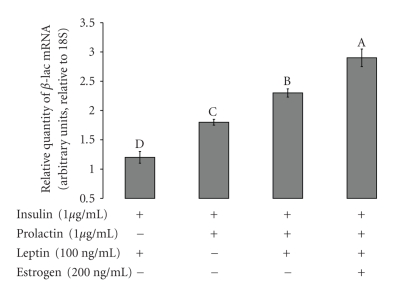
*Effects of leptin, prolactin, and estrogen on beta-lactoglobulin expression in explants from mammary glands of lactating cows.* Mammary-gland explants from lactating cows were incubated for 4 days in media containing various treatments: prolactin at 1 *μ*g mL^−1^, leptin at 50 ng mL^−1^, a combination of leptin and prolactin, and a combination of leptin and estrogen at 200 ng mL^−1^. Expression of the beta-lactoglobulin gene was evaluated by real-time PCR. Leptin alone elevated beta-lactoglobulin expression to a level of 1.2 arbitrary units, a level, which was not significantly different from that with insulin alone. Medium containing prolactin elevated beta-lactoglobulin expression to a level of 1.8 arbitrary units. When leptin and prolactin together were added to the medium, the level of beta-lactoglobulin reached 2.3 arbitrary units. Addition of estrogen to medium containing leptin and prolactin augmented the beta-lactoglobulin expression to 2.9 arbitrary units. Least squares means ± SE (*n* = 3). Bars with different letters differ at *P* < .05.

Mammary gland explants from lactating cows were incubated for 4 days in media containing various treatments of prolactin at 1 *μ*g mL^−1^, leptin at 50 ng mL^−1^, a combined treatment of leptin and prolactin, and a combined treatment prolactin at 1 *μ*g mL^−1^, leptin at 50 ng mL^−1^ with estrogen (at 200 ng/mL). The expression of beta-lactoglobulin was evaluated by real-time PCR. As shown in Figure 1, leptin alone had no effect on beta-lactoglobulin expression. Medium containing prolactin elevated the beta-lactoglobulin expression above the control level in a significant manner. Addition of leptin to medium containing prolactin up-regulated the lactogenic effect of prolactin. Addition of estrogen to medium containing leptin and prolactin significantly augmented the expression of beta-lactoglobulin to its highest level.

### 3.2. Expression of Aromatase mRNA in Mammary Fat Explants and Mammary Primary Culture

**Figure 2:**
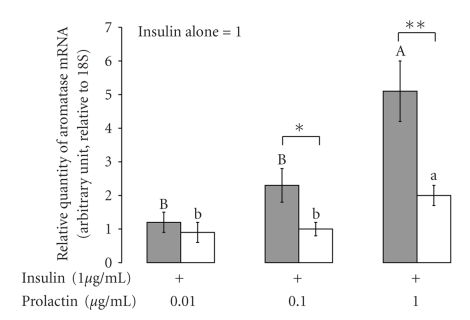
*Expression of aromatase mRNA in mammary fat explants and mammary primary culture.* Expression of aromatase mRNA in fat explants (closed bars) and primary mammary cells (open bars) from a lactating cow, with various concentrations of prolactin (0.01, 0.1 and 1 mg mL^−1^) in the medium. The expression of aromatase in cultures of primary cells did not change significantly. Significant expression of aromatase was observed in fat explants at prolactin concentrations of 1 mg mL^−1^. Least squares means ± SE (*n* = 3). Bars with different letters differ at *P* < .05. ∗, ∗∗ means differing at *P* < .05 and *P* < .01, respectively, between treatments with mammary primary cells and fat explants.

The expression of aromatase mRNA in fat explants (closed bars) and primary mammary cells (open bars) from a lactating cow was analyzed by real time PCR (Figure 2). The co-culture of cells and fat were incubated with several concentrations of prolactin (0.01, 0.1, and 1 *μ*g mL^−1^). The expression of aromatase in a primary cells culture did not change significantly, but was significantly elevated in fat explants at prolactin concentrations of 1 *μ*g mL^−1^: by almost 5.5-fold, respectively, compared with the control (basal medium).

### 3.3. Real Time Analysis of ER Alpha in Primary Mammary Cells from Lactating Cow

**Figure 3:**
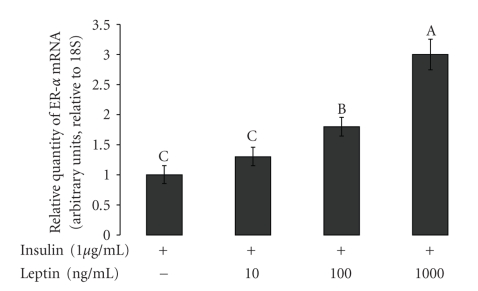
*Real time analysis of ER alpha in mammary explants from lactating cow after incubation with different levels of leptin.* Explants from a lactating bovine mammary gland were incubated with several concentrations of leptin (10, 100, and 1000 ng mL^−1^). ER alpha mRNA expression was up-regulated in a dose-dependent manner. At a leptin concentration of 10 ng mL^−1^ the expression of ER alpha reached a level of 1.3 arbitrary units, at 100 ng mL^−1^ leptin elevated ER alpha expression to 1.9 arbitrary units, and at a leptin concentration of 100 ng mL^−1^ the ER alpha expression level reached 3 arbitrary units. Least squares means ± SE (*n* = 3). Bars with different letters differ at *P* < .05.

Primary mammary cells from a lactating bovine mammary gland were incubated with different concentrations of leptin (10, 100, and 1000 ng mL^−1^) and one concentration of leptin antagonist (3200 ng ml^−1^). ER alpha mRNA expression was analyzed by real-time PCR. As shown in Figure 3, the expression ER alpha was up-regulated in the presence of leptin in a dose-dependent manner. Addition of leptin antagonist at a concentration of 3200 ng mL^−1^ to the medium containing leptin at 100 ng mL^−1^, down-regulated the expression of ER alpha.

### 3.4. Effect of Prolactin on ER Alpha Expression in Primary Cell Culture from Mammary Glands of Lactating Cows with or without Fat Explants

**Figure 4:**
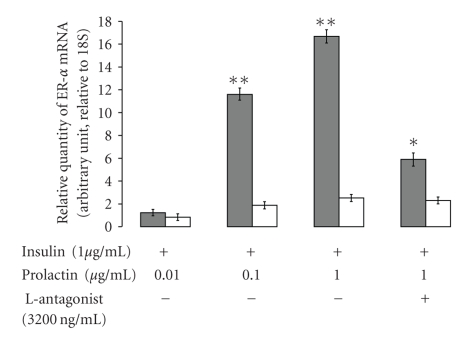
*The effect of fat explants on ER alpha expression in bovine mammary gland primary cell culture.* The expression of ER alpha was examined in primary culture that was incubated with mammary fat explants (closed bars) or without mammary fat explants (open bars). Four treatments were introduced: three different levels of prolactin (0.01, 0.1, and 1 *μ*g/mL) and one combined treatment of leptin antagonist (3200 ng/mL) and 1 *μ*g/mL prolactin. The expression of ER alpha in primary culture incubated with no fat explants did not change in the same magnitude when compared to explants incubated with fat explants. A significant difference in the expression of ER alpha between the treatments containing fat explants and those without was observed at prolactin concentrations of 0.1 and 1 *μ*g/mL prolactin. Leptin antagonist minimized the differences between the two treatments (with fat and without). Results are least square means ± S.E.M. of four independent experiments (*n* = 3). ∗, ∗∗ means differing at *P* < .05 and *P* < .001, respectively, between treatments with and without fat-explants.

Primary mammary cell culture and mammary fat explants were incubated with several concentrations of prolactin (0.01, 0.1 and 1 *μ*g mL^−1^), and 3200 ng/mL leptin antagonist in medium containing 1 *μ*g mL^−1^ prolactin. The primary culture was incubated with or without mammary fat explants. The expression of ER alpha in bovine mammary gland primary culture is shown in Figure 4. The addition of prolactin to the medium elevated the level of ER alpha mRNA expression in mammary cells incubated with mammary fat explants. A level 16 arbitrary units of ER alpha mRNA expression in the mammary cells was observed at the prolactin concentration of 1 *μ*g mL^−1^. Leptin antagonist down regulated the enhancement of ER alpha expression to a level of 6 arbitrary units of ER alpha mRNA expression. The expression of ER alpha in primary culture that were not incubated with fat explants did not change dramatically (0.8, 1.8, 2.5, and 2.3 arbitrary units of ER alpha mRNA expression at prolactin concentration of 0.01, 0.1, 1 *μ*g mL^−1^ in the medium, and 1 *μ*g/mL and 3200 ng/mL leptin antagonist, resp.). Throughout the experiment, ER alpha expression was robust in the treatments containing fat explants relative to the primary culture alone (basal medium).

### 3.5. Effects of Prolactin and Leptin on Cholesterol Secretion to the Culture Medium of Mammary Explants

**Figure 5:**
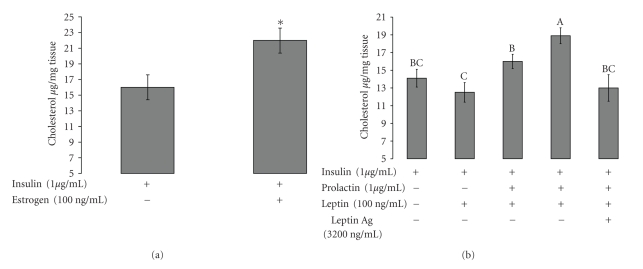
*Cholesterol secretion to the medium by mammary gland explants.* (a) Effect of estrogen on cholesterol secretion to the medium from mammary explants culture. Bovine mammary explants from a lactating cow were incubated in medium containing insulin at 1 *μ*g mL^−1^, with or without estrogen at 200 ng mL^−1^. The secretion of cholesterol to the medium in which explants were incubated with insulin was 14.5  *μ*g mL^−1^ per milligram of tissue; addition of estrogen to the medium containing insulin elevated the cholesterol secretion to level of 20 *μ*g mL^−1^ per milligram of tissue. Results are least squares means ± SE (*n* = 4). Bars with different letters differ at *P* < .05. (b) Effects of prolactin, leptin, and leptin antagonist on cholesterol secretion to the medium from mammary explants culture. Bovine mammary explants from a lactating cow were incubated in medium with or without prolactin at 1 *μ*g mL^−1^, with or without leptin at 100 ng mL^−1^, or with a combination of leptin at 100 ng mL^−1^ and prolactin at 1 *μ*g mL^−1^, with or without leptin antagonist. Addition of leptin to the medium did not affect the secretion of cholesterol to the medium. Prolactin at a concentration of 1 *μ*g mL^−1^ elevated the secretion of cholesterol 16 *μ*g mL^−1^ per milligram of tissue. A combination of leptin at 100 ng mL^−1^ and prolactin at 1 *μ*g mL^−1^ elevated the secretion of cholesterol to its highest level of 19 *μ*g mL^−1^ per milligram of tissue. Addition of leptin antagonist to the leptin plus prolactin combined treatment down-regulated the secretion of cholesterol to 13.2 *μ*g mL^−1^ per milligram of tissue. Results are least squares means ± SE (*n* = 3). Bars with different letters differ at *P* < .05.

Mammary explants from a lactating cow were incubated in basal medium with or without estrogen at 100 ng mL^−1^. Addition of estrogen elevated the secretion of cholesterol from the tissue to 20 *μ*g mL^−1^ (Figure 5(a)). Addition of leptin to the basal medium did not affect the secretion of cholesterol to the medium. Prolactin elevated the secretion of cholesterol to 16 *μ*g mL per mg of tissue and the combination of leptin at 100 ng mL^−1^ and prolactin at 1 *μ*g mL^−1^ elevated the secretion of cholesterol to its highest level of 19 *μ*g mL^−1^ per milligram of tissue. Addition of leptin antagonist to the combined treatment of leptin and prolactin down-regulated the secretion of cholesterol to 13.2 *μ*g mL^−1^ per milligram of tissue (Figure 5(b)).

## 4. Discussion

The role of estrogen during bovine mammogenesis has been well established [8]. Estrogen is essential for mammary epithelial proliferation and differentiation [9]. Janowski et al. [33], examined the mammary secretion of estrogen to the mammary vein in lactating cows, found that around parturition the plasma concentration of estrogen in the abdominal vein was significantly higher than that in the peripheral blood. They also showed that lactating bovine mammary tissue could convert andostendione to oestradiol-17 beta; their findings clearly showed that the mammary gland could produce and secrete estrogen, and that the level of estrogen secretion from the mammary gland is correlated with the levels of prolactin around parturition. Moreover, Janowski et al. [33] found a correlation between the level of estrogen secretion and high-yielding cows, which clearly suggests that estrogen has a role in the regulation of lactation. In the present study, we showed that estrogen affected beta-lactoglobulin expression in bovine mammary explants, and that addition of estrogen to medium containing prolactin and leptin up-regulated the expression of beta-lactoglobulin in mammary explants to a higher level than that in explants incubated with leptin and prolactin (Figure 1). These findings are consistent with those of Janowski et al. [33] regarding the lactogenic effect of estrogen on bovine lactation. Previously, [20], we demonstrated that leptin had the ability to up-regulate the lactogenic action of prolactin in the lactating mammary gland. We also demonstrated that the mammary fat has an essential role in the regulation of leptin stimuli on prolactin lactogenic effect [28]. Since, we believe that estrogen in general, and mammary fat-derived estrogen in particular, is involved in the regulation of bovine lactation, we examined the expression of aromatase mRNA in bovine mammary primary cells and bovine mammary fat. We showed that prolactin could regulate aromatase mRNA expression in mammary fat pad. We also show that prolactin at a level of 1 *μ*g/mL can elevate the aromatase expression in bovine mammary primary cell cultures (Figure 2). In light of the present findings, it is suggested that prolactin regulates estrogen secretion from the mammary gland and from the mammary fat in particular. 

Leptin is one of the factors that are secreted from the mammary fat tissue. We hypothesize that the mammary fat-derived leptin can regulate the action of estrogen on the bovine mammary gland. Therefore, we examined the effect of leptin on ER alpha, and found that leptin regulated its' expression in bovine mammary primary cells (Figure 3). Earlier, we demonstrated the positive effect of prolactin stimulation of mammary fat explants on alpha casein expression in bovine mammary explants and leptin secretion from mammary fat explants [28]. Mammary fat explants in the presences of prolactin up regulated the expression of ER alpha in primary cell culture (Figure 4). When fat explants were not present it the culture the expression of ER alpha did not change dramatically compared to the incubation with fat explants. Connor et al. [11] showed that ER alpha appeared to be solely expressed in the mammary epithelial cells. Our present finding, that leptin antagonist down regulated the expression of ER alpha in mammary primary cells (as shown in Figure 4) supports our hypothesis that leptin regulates ER alpha expression. These findings are supported by those of Catalano et al. [34], who demonstrated that leptin is capable to up-regulate the expression of ER alpha in MCF-7 cells. We believe that the prolactin peak around parturition induces the secretion of leptin and estrogen from the mammary gland fat. Mammary-derived leptin activates the ER alpha expression in the mammary gland, and thus regulates the action of estrogen on the mammary gland. Studies which examined the levels of leptin around parturition showed that the concentration of leptin [35] and leptin receptor [19] decreased near and after parturition. These findings might contradict our hypothesis, however, we believe that the level of leptin in the peripheral blood does not reflect the activity of leptin within the secretory mammary units. Moreover, the expression of leptin receptor in ruminants after parturition was expressed mainly in the mammary alveolar epithelial cells [19], which clearly suggests that leptin has a regulatory effect on the alveolar unit. The notion that leptin acts at the autocrine level is supported by studies such as that of Silva [36], which showed that the bovine mammary gland expresses the long form of the leptin receptor (OB-Rb), and claimed that the local concentration of leptin in the pre-pubertal mammary gland might not be reflected in the leptin concentration in the circulation. Additional support can be found in our previous study [28], in which we demonstrated that addition of leptin antagonist to an explants culture, that was incubated only with prolactin, down-regulated the expression of beta-lactoglobulin. This finding clearly indicates the involvement of prolactin in the regulation of mammary-derived leptin and leptin receptor in the bovine mammary explants. We examined the effect of estrogen on the secretion of total cholesterol from explants culture and found that estrogen, with or without prolactin, up-regulated the secretion of cholesterol from lactating mammary gland explants to the medium (Figure 5(a)). We also showed that prolactin could up-regulate the secretion of cholesterol by the mammary explants and that addition of leptin elevated the cholesterol secretion to the medium. Leptin antagonist eliminated the effect of leptin on cholesterol secretion by the mammary explants (Figure 5(b)). Previously, we have demonstrated that leptin and prolactin up regulated the lactogenic markers such as fatty acid synthesis and milk protein synthesis [20], therefore it is not surprising that leptin and prolactin up regulated the total cholesterol secretion from the bovine mammary gland explants.

## 5. Conclusion

Based on all of the above we believe that the prolactin peak around parturition activates an epithelial/mammary fat interaction, which can affect the potential milk yield. based on all of the above, we suggest that the level of leptin, which is correlated with the mammary fat mass, affects the expression of ER alpha in the epithelial mammary cells and thereby regulates the effect of mammary-derived estrogen on the mammary gland.
